# “Post partum hemorrhage: causes and management”

**DOI:** 10.1186/1756-0500-6-236

**Published:** 2013-06-18

**Authors:** Muhammad Muzzammil Edhi, Hafiz Muhammad Aslam, Zehra Naqvi, Haleema Hashmi

**Affiliations:** 1 Liaquat National Medical College, 402, Al Jannat Plaza, M.A. Jinnah road Saed manzil, Karachis, Pakistan; 2 Dow Medical College (DUHS), Flat #14, 3rd floor, Rafiq Mansion, Cambell road, Off Arambagh, Karachi, Pakistan; 3 Obstetrics and Gyneacology Department, Liaquat National Medical college, Liaquat National Hospital, Karachi, Pakistan

**Keywords:** Postpartum hemorrhage, Uterine atony, Hysterectomy

## Abstract

**Background:**

Post partum hemorrhage is defined as blood loss of 500 ml or above. It is the most common cause of pre-mature mortality of women world wide. Our objective was to evaluate the most common etiology and method of management of Post partum Hemorrhage in a tertiary care hospital of Karachi.

**Findings:**

It was a cross sectional study conducted at Liaquat National Hospital Karachi, during the period of July 2011 to May 2012. Review include mode of delivery, possible cause of postpartum hemorrhage, supportive, medical and surgical interventions. All the women admitted with post partum hemorrhage or develop PPH in hospital after delivery were included in our study. Bleeding disorder and use of anticoagulants were set as exclusion criteria. Diagnosis was made on the basis of blood loss assessment which was made via subjective and objective evaluation.

During the targeted months, out of total 1493 deliveries (26/1493 = 1.741%) 26 cases of post partum hemorrhage were reported with a mean age of 26.153 ± 7.37. No deaths were reported and all cases were referred and unbooked cases. All Patients were conscious, tachycardiac and hypotensive. Most of the women were suffering from hemorrhage during or after the birth of their 1st child. Primary post partum hemorrhage emerge as the most common type of post partum hemorrhage and uterine atony was detected as the most common cause of primary post partum hemorrhage. Retained products of conception was the most common cause of secondary post partum hemorrhage and hysterectomy was found to be the most frequent method of management of post partum hemorrhage.

**Conclusion:**

This study highlights the existing variable practices for the management of postpartum hemorrhage. Hemorrhage associated morbidity and mortality can be prevented by critical judgment, early referral and resuscitation by attendants. Introduction of an evidence-based management model can potentially reduce the practice variability and improve the quality of care.

## Findings

### Background

Death due to pregnancy remains an important cause of premature mortality of women worldwide, an estimated 500,000 women die from this cause every year with up to quarter of deaths occur due to hemorrhage [1]. Post Partum hemorrhage may occur in 1-5% of deliveries in developed as well as in developing countries and it is still most common cause of maternal morbidity and mortality [2]. It is axiomatic that Post partum hemorrhage occur unpredictably and no patient is immune from it, it simply states that it is an equal opportunity killer. Blood loss of 500 ml following a delivery is generally considered as physiologically normal and anything above this limit is known as Post partum hemorrhage [3]. For vaginal delivery blood loss of above 500 ml and in C-section blood loss of above 1500 ml. Another definition of PPH is that blood loss sufficient to cause hypovolemia, a 10% drop in the hematocrit or requiring transfusion of blood products (regardless of route of delivery) [2]. Prevalence of death due to PPH in Pakistan is 34% [4].

Post partum hemorrhage was classified into two types, primary and secondary, Primary is defined as blood loss of greater than 500 ml due to vaginal delivery and loss of 1500 ml due to C section within first 24 hours of delivery [4,5]. Its incidence is 5% of all deliveries [5]. Secondary is defined as excessive vaginal blood loss or heavy lochial discharge occurring at least 24 hours after the end of the third stage of labor [6].

In many International and local studies it was revealed that the main cause of PPH is uterine atony followed by vaginal hematoma, cervical or vaginal tear, adherent placenta, uterine angle extension and retained placenta [3,5]. It is also indicated that massive PPH leads to complication like hypovolumic shock, disseminated intravascular coagulation ,hepatic dysfunction, acute respiratory distress syndrome and renal failure [3,5,6]. In order to prevent these complications, an organized stepwise protocol should be followed and active management of third stage of labor should be offered to all women.

Our basic aim was to evaluate and investigate the most common case of PPH in our system. It was also one of the main objectives to separately study the management and co morbid of patients of primary and secondary PPH.

### Methodology

It was a cross sectional study conducted on the diagnosed patient of postpartum hemorrhage which came to the Liaquat National Hospital; well known tertiary care hospital of Karachi, during the period of July 2011 to July 2012. During the targeted months, 26 cases of post partum hemorrhage were came in Liaquat National Hospital, after the arrival of each patient all four researchers were perform interview of patient and take her complete biodata and then take blood pressure and pulse of her. All information was gathered by taking written informed consent from patient itself and also verbal consent from attendants, which were her relatives. Surgical intervention, mode of delivery and management method was noted from the medical record files and surgical notes of the patients. Criteria for the diagnosis of PPH was bleeding >500 ml following vaginal delivery or >1500 ml following C-section. Criteria for the diagnosis of Primary Post partum hemorrhage was that if blood loss occur within 24 hour then it was considered as primary post partum hemorrhage and if it occurs beyond 24 hour to 6 weeks then it was considered as secondary postpartum hemorrhage. Blood loss was measured from the time of delivery until the mother was transferred to postnatal care. Immediately after the cord was clumped out, the blood collection was started by passing a flat bedpan under the buttocks of a women delivering in a bed for a women delivering on a delivery table. Blood collection and measurement continued until the third stage of labor was completed and the woman was transferred to the postnatal ward. At that time, the collected blood was poured into a standard measuring jar and its volume measured. To simplify the procedure for measurement of blood loss, any available small gauze swabs soaked with blood were put into the measuring jar and included in the measurement together with the blood and clots; along with the objective assessment of drop in hemoglobin levels and need for blood transfusion [7]. Pre- Haemoglobin was measured at the time of admission. Post - Hb% was done 48 hours after the delivery. All women admitted with post partum hemorrhage or develop PPH in hospital after deliveries were included in study. Exclusion criteria were patients with history of bleeding disorders and those on anticoagulants. Pulse rate was measured through the standard method of pulse rate counting and Blood pressure was taken with the aneroid manometer. Information was collected through proper and structured questionnaire, which was developed according to the need of study. Questionnaire comprises of the questions on Biodata, status of delivery, mode of delivery, possible causes of PPH, reading of pulse rate and blood pressure, diagnosing procedure, supportive, medical, and surgical interventions [Additional files 1 and 2]. Study was ethically approved by Ethical Review Committee of Liaquat National Medical College, Liaquat National Hospital. All the data was entered in SPSS 19 and also analyzed through it. Chi-square test was used for the evaluation of difference of median of type of post partum hemorrhage and the causes and management of post partum hemorrhage and their percentages were calculated by using cross Tabs in such a way that each column add up to 100% in the variables of classification of post partum hemorrhage, mode of delivery, status of delivery, place of delivery, blood pressure, pulse rate, comorbids and parity. While each row add up to 100% in the variable of causes and methods of management of Post partum hemorrhage. The threshold for statistical significance was set at P < 0.05. Rests of the frequencies were calculated from simple frequency table.

### Result

There were total 26 patients of postpartum hemorrhage came in Liaquat National Hospital in our selected time period of study. Total number of deliveries occured in liaquat national hospital during July 2011 – May 2012 were 1493,therefore Post partum hemorrhage occured in 1.7% (26/1493 = 1.7%) cases. No deaths were reported and patients were between the ages of 19–45 with a means age of 26.153 ± 7.37.

All cases were unbooked and fully conscious. There were 24(92.3%) cases which referred from local hospitals and 2(7.7%) referred from other tertiary care hospitals.

Most of the patients had post partum hemorrhage after the delivery of 1st child 10(38.5%), while 8(30.8%) suffered after the birth of 3rd child and 4(15.4%) suffered after the birth of 2nd child (Table 1).

**Table 1:** Table represents the biodata of patients

**S. no**	**Variables**	**Frequency (n)**	**Percentage (%)**
1	**Classification of post partum hemorrhage**		
a	Primary post partum hemorrhage	**18**	**69.2**
b	Secondary post partum hemorrhage	8	30.8
2	**Mode of delivery**		
a	Spontaneous vaginal delivery	**16**	**61.5**
b	C-section	10	38.5
c	Episiotomy in SVD patients.	6	23.1
3	**Status of delivery**		
a	Book	0	0
b	Unbook	**26**	**100**
4	**Place of delivery**		
a	Local hospital	**24**	**92.3**
b	Tertiary care hospital	2	7.7
5	**Blood pressure**		
a	Hypertension	0	0
b	Normal	0	0
c	Hypotension	**26**	**100**
6	**Pulse rate**		
a	High	**26**	**100**
b	Normal	0	0
c	Low	0	0
7	**Comorbids**		
a	Hepatitis	4	15.4
b	Eclampsia	4	15.4
c	Not any	**18**	**69.2**
8	**Parity**		
a	Parity 1	**10**	**38.5**
b	Parity 2	4	15.4
c	Parity 3	8	30.8
d	Parity 3 + miscarriages 2	2	7.7
e	Parity 3+ miscarriages 1	2	7.7

Total no of deliveries (n = 1493) and total cases of PPH (n = 26).

• Highest values were indicated in bold.

• Columns of each variable add up to 100%.

Most common mode of delivery in patients of post partum hemorrhage were Spontaneous vaginal delivery 16(61.5%) followed by C-section 10(38.5%) and in 6(23.1%) had episiotomy with spontaneous vaginal delivery. All patients suffered from tachycardia as well as from hypotension. Most of the patients had no co-morbid while 4(15.4%) had Hepatitis and 4(15.4%) had eclampsia (Table 1).

There were 18(69.2%) patients who had primary Post Partum Hemorrhage while 8(30.8%) were suffered from secondary Post Partum Hemorrhage .Most common cause of Primary Post Partum Hemorrhage was Uterine atony which was found in 6 patients (p = 0.68) followed by cervical and vaginal tear in 6(p = 0.677) patients, uterine inversion in 2(p = 0.336) patients, Morbidity adherent placenta 2(p = 0.336) patients, and placenta previa in 2(p = 0.336) patients. Most frequent cause of Secondary post partum hemorrhage was Remaining products of Placenta which was found in 8(p = <0.01) patients. Out of total 8 patients with cause of remaining products of conception, 2(p = 0.67) also had the cause of cervical or vaginal tear (Table 2).

**Table 2:** Table represents the causes of post partum hemorrhage

**S. no**	**Causes**	**Primary post partum hemorrhage n (%)**	**Secondary post partum hemorrhage n (%)**	**P-value**
1	Uterine atony	**6(100)**	0	**0.63**
2	Remaining products of conception	0	**8(100)**	**<0.01**
3	Cervical or vaginal tear	**6(75)**	2(25)*	**0.671**
4	Morbidity adherent placenta	**2(100)**	0	**0.326**
5	Placenta pravia	**2(100)**	0	**0.326**
6	Uterine inversion	**2(100)**	0	**0.326**

Total no of deliveries (n = 1493) and total cases of PPH (n = 26).

• Threshold for significance is 0.05.

• Highest values indicated in Bold.

• Results were evaluated by considering the group of primary and secondary post partum hemorrhage as 100%.

• Patients also had multiple causes of Post partum hemorrhage.

• Rows of each variable add up to 100%.

* Out of 8 patients with cause of remaining products of conception 2 have cervical or vaginal tear.

There were different management procedures adopted for the treatment of Primary or Secondary Post Partum Hemorrhage. Most reported method for the management of Primary Post Partum Hemorrhage was Hysterectomy, done in 8(p = 0.026) patients followed by Repair of cervical and vaginal tear in 6(p = 0.677) patients, Internal iliac ligation in 2(p = 0.336) and replacement of uterine inversion in 2 (p = 0.505) patients (Table 3).

**Table 3:** Table represent the methods of management of post partum hemorrhage

**S. no**	**Methods of management**	**Primary post partum hemorrhage n (%)**	**Secondary post partum hemorrhage n (%)**	**p-value**
1	Evacuation of remaining products of conception	0	**8(100)**	**<0.01**
2	Repair of cervical and vaginal tear	6(75)	2(25)*	**0.671**
3	Internal iliac ligation	2(100)	0	**0.326**
4	Hysterectomy	**8(100)**	0	**0.023**
5	Replacement of uterine inversion	1(100)	0	**0.497**

Total no of deliveries (n = 1493) and total cases of PPH (n = 26).

➢ Threshold for significance is 0.05.

➢ Highest values indicated in Bold.

➢ Results were evaluated by considering the group of primary and secondary post partum hemorrhage as 100%.

➢ Multiple methods of management also adopted in same patient.

➢ Rows of each variable add up to 100%.

*Method of management use with evacuation of remaining product of conception.

Commonest management methods used for the treatment of Secondary Post partum Hemorrhage were Evacuation of remaining products of conception which was adopted in 8(p = <0.01) patients. Out of 8 patients of secondary post partum hemorrhage, 2(p = 0.67) also had cervical or vaginal tear and for their management two methods of management were used, evacuation of remaining products of conception and repair of cervical or vaginal tear (Table 3).

### Discussion

Post partum hemorrhage is the leading cause of morbidity and mortality among pregnant ladies throughout the world causing 140,000 deaths each year globally, this corresponds to the one women dying in every 4 minutes and it is the 5th most common cause of maternal mortality throughout the world [3,8-12].

It was found in our study that all cases were either referred from primary or tertiary care hospitals and there were no home deliveries. There were no booked cases and also no deaths were reported during the targeted tenure of study; these findings were comparable with the findings in previous studies [3,8].

It was interestingly noted that all women were conscious, hypotensive and tachycardiac, these findings were consistent with the another study occur on the same topic [8]. This also supported the fact that blood loss results in tachycardia and a measurable fall in the blood pressure [13].

Commonest mode of delivery which was found in our setup was Spontaneous vaginal delivery followed by C-section, while nearly half of the cases of spontaneous vaginal delivery undergo episiotomy. Result was nearly same as shown in previous studies occur in Pakistan in 2006 and 2011 respectively [3,5].

Most important and major finding in our study was that the most common cause of Post partum hemorrhage was uterine atony, which is loss of tone in the uterine musculature. Normally, contraction of the uterine muscle compresses the vessels and reduces flow. This increases the likelihood of coagulation and prevents bleeds. Thus, lack of uterine muscle contraction can cause an acute hemorrhage. These findings were evident by the studies conducted in America and Pakistan [3,5,12,14]. Other causes which were responsible for this lethal condition were uterine inversion, which is a potentially fatal childbirth complication with a very low maternal survival rate and occurs when the placenta fails to detach from the uterus as it exits, pulls on the inside surface, and turns the organ inside out and rest of the causes are cervical or vaginal tear, morbidity adherent placenta, an abnormality in the adherence of the placenta to the myometrium, and placenta previa as shown in previous studies [3,5,12,14].

Statistical analysis of different causes of secondary post partum hemorrhage portrayed that the most common cause of secondary post partum hemorrhage was retained uterine products same as found in past studies [2,6]. Cervical and vaginal tear also emerged as one of the cause of delayed or secondary post partum hemorrhage in contrast to the past studies [2,6,13].

In our setup of tertiary care we used Evacuation of remaining products of conception and hysterectomy, surgical removal of the uterus, as the principal tools for the management of post partum hemorrhage as indicated in another studies [2,3,5]. While other methods were repair of cervical and vaginal tear and replacement of uterine inversion [2,3,5,12,14].

Our study was cross sectional study encircling a small part of population of Karachi and may not be representative of whole scenario of city. Liaquat National hospital is one of the luxirious hospital of karachi which is affordable only by high and high middle class i.e. result mostly represents the condition of upper class not all the socioecnomic classes of city. For the better understanding of disease it is very necessary to do a large scale study covering the major part of population. Post partum hemorrhage is one of the top most causes of maternal mortality in our setup and it is very necessary to take steps to reduce the morbidity and mortality. It’s also necessary for every health care setup to correctly asses the blood loss following delivery and then manage the patient accordingly.

### Conclusion

It is defined that PPH is the leading cause of maternal morbidity and mortality. In order to prevent this fatal disease it is very essential to accurately assess the risk factors and blood loss during delivery. It is also recommended to strictly follow the authorized management plan in order to prevent the patient with complications and death. It should be emphasized that all patients should receive active management for the 3rd stage of labor. Additionally, strict adherence to formulated protocols and guidelines is important to further improve outcomes in patients with massive PPH. Lastly, more studies like the one we performed are important to recognize and rectify any deficiencies in obstetrical practice.

## Abbreviations

PPH: Post partum hemorrhage; C-section: Cesarean section.

## Competing interests

The authors declare that they have no competing interests.

## Authors’ contributions

MME, HMA, did data collection, analyzing and manuscript drafting while ZN and HH did critical review of manuscript. All authors give approval to the final version of manuscript.

## Supplementary Material

Additional file 1 Questionnaire page 1. Click here for file

Additional file 2 Questionnaire page 2. Click here for file
