# Supplementary figures and images for: “Post partum hemorrhage: causes and management”
Source: BMC Res Notes. 2013 Jun 18;6:236. doi: 10.1186/1756-0500-6-236 (PMC3688110; doi:10.1186/1756-0500-6-236)

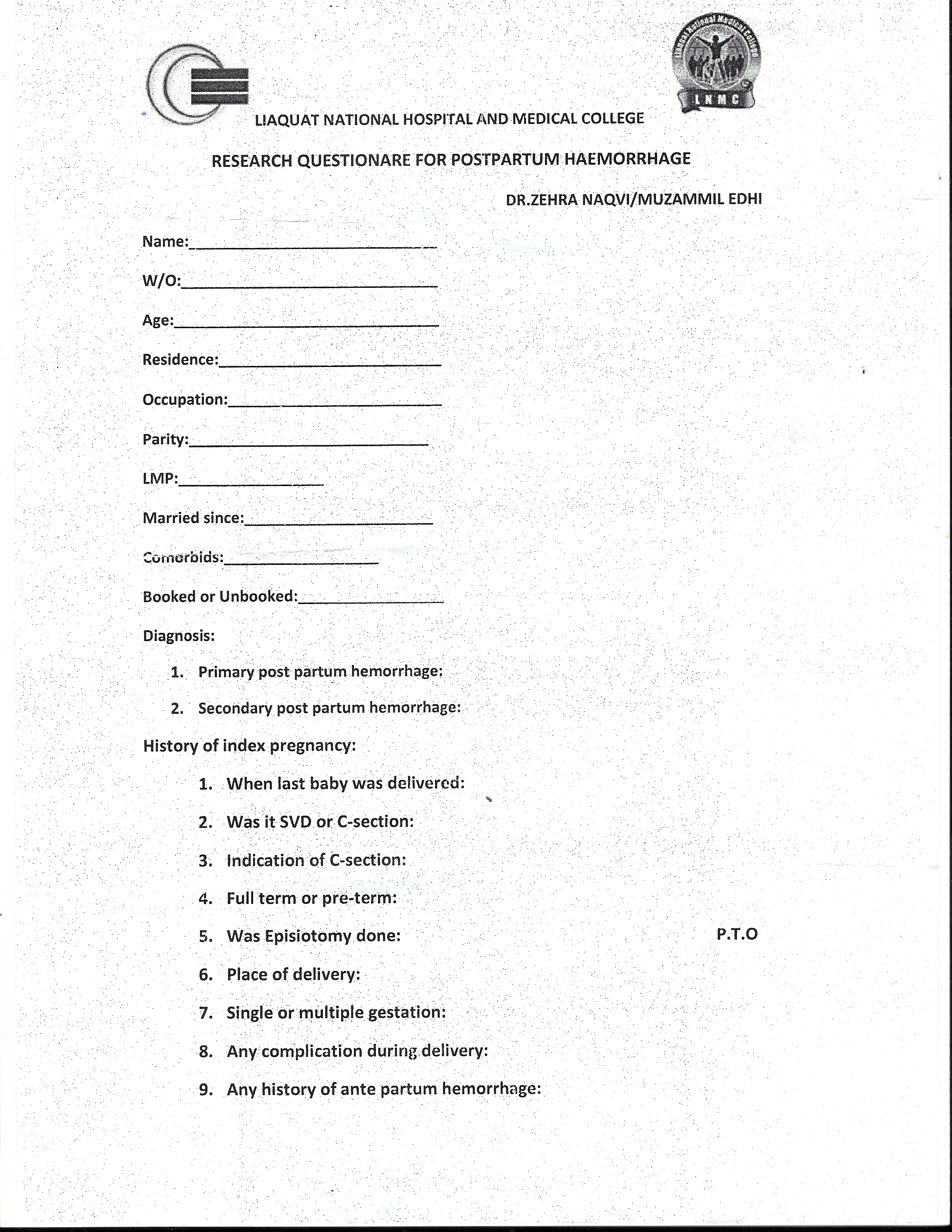

Supplement: Additional file 1 — Questionnaire page 1. [file 1756-0500-6-236-S1.jpeg]

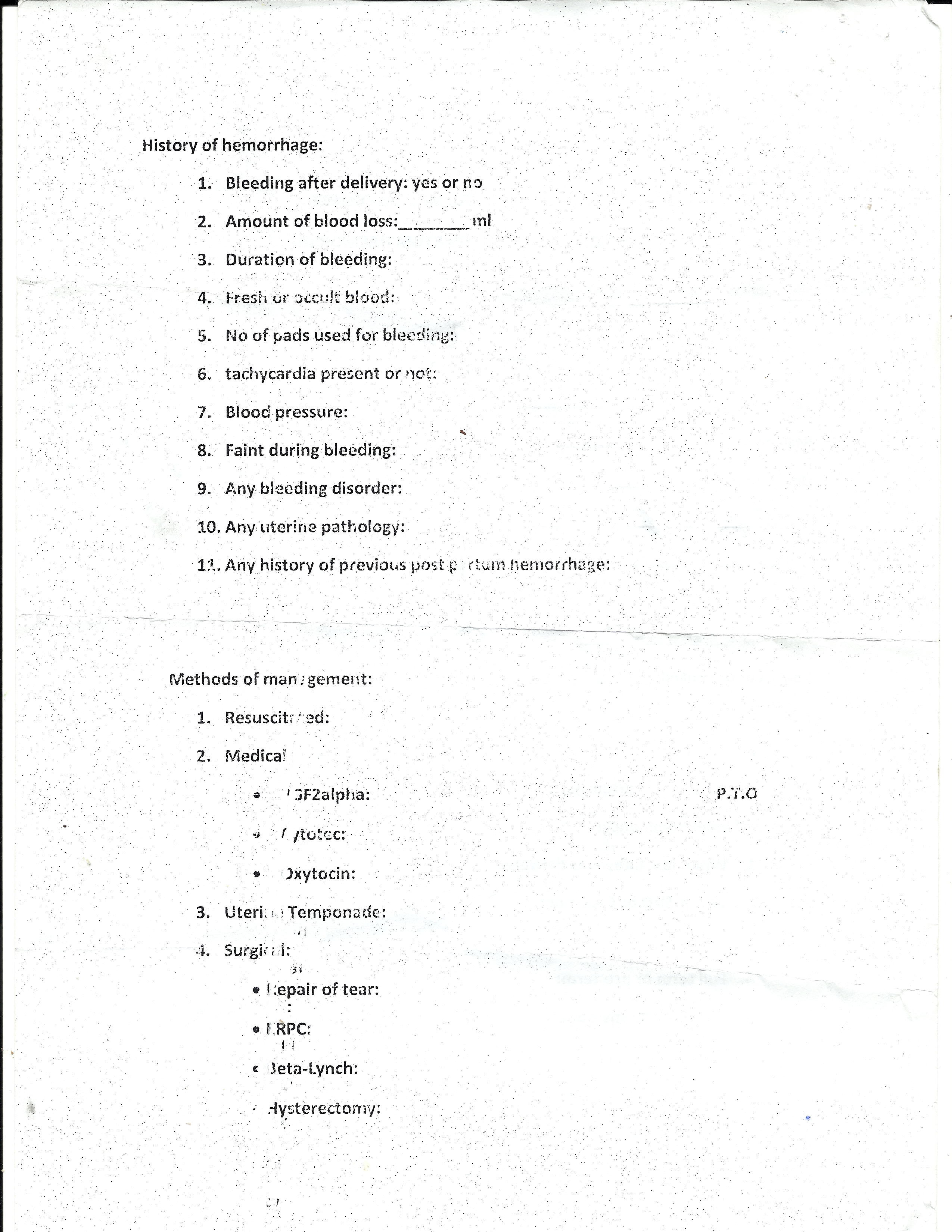

Supplement: Additional file 2 — Questionnaire page 2. [file 1756-0500-6-236-S2.jpeg]
